# Notes and News

**Published:** 1889-08-31

**Authors:** 


### NOTES AND NEWS.

COLLECTED AND COMMUNICATED.

THE National Home Reading Union has now opened its office in Surrey House, Victoria Embankment. Miss Mondy has been appointed secretary of the young people's section, and Mr. George Howell of the artisan's department.

THE papers read at the British Dental Association were interesting, especially one on anæsthetics. Sir John Tomes, Sir Edwin Saunders, and Mr. John Smith (Edinburgh), were elected vice-presidents of the association. It was decided to hold the next annual meeting at Exeter, Mr. Browne-Mason, of that city, being chosen president-elect.

DR. COLLINSON, whose term of office at the Durham County Hospital has recently expired, was last week presented by the nursing staff with a handsome testimonial, consisting of a set of brass ornaments for the writing table, in recognition of their appreciation of the harmonious manner in which they had worked together during his residence as house surgeon.

THE annual meeting of Ancoats Hospital, Manchester, was held on August 15, Mr. Jardine presiding. The visit of Prince Albert Victor, who laid the foundation-stone of the new wing last October, drew special attention to the work and the needs of the hospital; but the committee, whilst gratefully acknowledging the response that has been made to their appeal, are still in need of money. During the year 4,487 "accidents" have been treated, and altogether some 10,169 patients have benefited by the existence of the hospital. Mr. Heywood, in an excellent speech, spoke of the growth of medical charities in Manchester. Four conspicuous and important hospitals had been added since 1870—viz., the Hospital for Incurables, the Ancoats Hospital, the Hospital for Consumption, and the Victoria Dental Hospital. The fourteen hospitals existing in 1870 provided for 508 patients, while the eighteen hospitals existing now provided for 1,240.

LAST Saturday a delightful trip was arranged to Morley House Home. The committee of this institution, of which the Earl of Aberdeen is president, arranged for a cheap excursion to St. Margaret's Bay, Dover, which is in the vicinity of the home. A long list of attractions was provided, including open-air concert, handbell ringing, dancing, athletic sports, etc. The weather was not quite all that was desired, but the arrangements passed off successfully.

IN aid of the Barnsley Beckett Hospital and Dispensary two demonstrations have been held. At Wombwell a choir of 200 voices performed Handel's "Samson" in the open air on Sunday afternoon, and the Rev. G. Hadfield spoke in favour of charity to the sick. At Monk Bretton a sacred concert was also held in the open air, and several ministers spoke. The effect was rather incongruous, as some of the members of the Foresters' Society had come dressed as Friar Tuck, Robin Hood, etc.

A MEETING of the representatives of trades' unions in Barrow has been held to take into consideration the desirability of taking steps to secure direct representation on the Hospital Council of men of their own class; also to take means to secure more direct control of the collection of subscriptions for the support of that institution. It is a great mistake to try and keep working-men out of the committees of hospitals, and we sincerely hope the Trades' Council will get two representatives on the board shortly.

MISS FRANCES POWER COBBE is not loved in medical circles because she is a lady who is devoted to fads. Still there is some truth in a recent sentence of hers anent hospitals: "As regards the sacred institution of the family, on which society itself is based, I ask what, except evil, *can* result from the habitual separation of the relatives the moment that illness makes a claim for tenderness and care?" Hospitals are meant for accidents, operation cases, and severe medical cases, but alas! they are too often used as receptacles for the miscellaneous sickly. We deeply regret it, and venture to think that the fault lies with the careless distribution of patients' tickets. Hospital physicians are not always prowling about the streets seeking for cases to carry off to their wards, as Miss Cobbe seems to think.

MR. GEORGE CARSON, of Leeds, is to be the architect for the new wing of the infirmary in that town. The extension is to include special and administrative departments. It will be heated by steampipes, supplied from present boilers, and arranged in sections, with valves to each section, so that they can be opened, shut, or regulated independently. The provision for ventilation is to be of the most ample nature. The open fireplaces in the wards will, as in the present building, assist in drawing off the foul air; but, in addition, it is proposed to form horizontal flues in the floors between the joists, opening with gratings in the walls at each end, and with upcast shafts in the ceilings, with arrangement of gas jets to assist the current. Where the roof is immediately over the room these horizontal flues would be formed in it, and have gablets at each end. The estimated cost of the new building is £17,000. The walls will be of brick, faced with pressed brick, and dressed with Bolton Wood stone. The roofs will be constructed of Memel timber, and covered with 2in. boarding and dark Westmoreland slates. The floors of the wards will be laid with oak boards, and those of the waiting-rooms with solid herring-bone oak flooring. The walls of the isolation-room and the isolation wing will be lined with glazed bricks of cream colour. The bathrooms, etc., will be similarly lined, and the waiting-rooms and corridors will be lined to the height of 6ft., and will have a base and a surbase of moulded or glazed terra-cotta.

The following vacancies are announced this week, the amount of the salary in each case being given after the name of the institution:

**Resident Medical Officers.**

Borough Hospital, Birkenhead. Junior House Surgeon.—£60, board, &c.

Birmingham Hospital. Surgical Officer.—£130, board, &c.

Wallasey Dispensary. House Surgeon.—£110, residence, &c.

Stamford Infirmary. House Surgeon and Secretary.—£100, board, &c.

Wolverhampton Hospital. House Physician. — £100, board, &c.

Rotherham Hospital. Assistant House Surgeon.—Board, &c.

North London Consumption Hospital. Medical Officer.—£40, board, &c.

Swansea Hospital. Medical Officer.—£100, board, &c.

The public journals tell of more miracles worked at Notre Dame de Lourdes. A young girl from Alençon, who had been blind for two years, recovered her sight when taken into the grotto. A navvy from Chartres received a hurt which paralysed him and made him a cripple for years. He instantly recovered the use of his limbs. We hope the use of the limbs will remain; sudden excitement is apt to work only temporary miracles.

The annual meeting of Lloyd Cottage Hospital was lately held, the Rev. Y. Lloyd Graeme presiding. The hospital does a good work, and quite lately every bed was occupied. The in-patients numbered 88, the out-patients 120 during the year; a considerable increase on last year's numbers. The hospital still has a balance of over £100 at the bank. Dr. G. R. Nelson has been appointed an honorary medical officer of the institution.

The disagreeing of doctors is as remarkable at Vienna as at Liverpool just now. Prince Sulkowski was pronounced mad by two doctors six years ago, and shut up in an asylum. Lately he escaped, and two other doctors now pronounce him sane. It is specially hard in the case of rich people who can afford to be eccentric to say whether they are actually insane or not. Many of us if we had no work to do and plenty of money to spend might like to keep a private menagerie.

Last Saturday afternoon H.R.H. Princess Frederica (Baroness Von Pawel Ramingen) presented prizes to the successful students of the ambulance classes held at the People's Palace, Mile End-road. The meeting was held in the Queen's Hall, and there was a very large attendance. The chair was occupied by Sir Vincent Kenneth Barrington, who was supported on the platform by Lady Barrington, Major Sir Herbert Parrott, Bart., Sir E. A. Currie, Rev. H. Jones, Colonel Aubone, and other ladies and gentlemen. The chairman gave a long address, in which he referred to the value of ambulance knowledge to police, railway porters, and other public servants. Several surgeons and physicians also spoke in favour of the association, and then Princess Frederica graciously distributed the prizes and certificates.

The annual meeting of the Dufferin Maternity Hospital, Burmah, was held at Government House, on June 14. The Chairman stated that the hospital and training institution so far had been successful. During the first year 142 passed through the hospital, in the second year 228, and up to date 405 women had been treated in the hospital, and there had been 209 births. Twenty women had this year passed as nurses, and fourteen of these as midwives. The experimental stage of the work was passed, and it had become absolutely necessary that a suitable building should be provided. The present structure is a temporary one, and may have to be vacated at any time. The Central Committee of India has contributed 5,000 rupees towards the building fund, and they themselves had set aside 5,000 rupees for the same purpose.

Dr. Richard Greene writes an article in the *Universal Review*, in which he declares that there is no such thing as monomania. Dr. Shaw, of Belmont Asylum, and others, have been heard to enunciate this same sentiment, and to say that a maniac is easily and rapidly recognised. Why then do we hear of such constant mistakes? Nothing is so common out of asylums as the belief that a great many lunatics are mad on one point only and sane on all others; and nothing is so rare as to find such cases in asylums.

At the last monthly meeting of Dublin Royal Hospital for Incurables, five new patients were admitted, of whom three—all men—were suffering from cancer of the tongue. It was reported that the "Victoria Jubilee Wing" had been handed over to the governors by the contractors last month, and that there are 91 patients now in occupation of the four bright and cheerful wards constituting the new wing, in which every modern improvement which can in any way ameliorate the condition of the inmates has been adopted.

Dr. Newington gave an interesting address before the Medico-Psychological Association on July 25th. He vigorously contested the depreciatory account that had been given of the progress in the study of insanity during past years. From the point of view of the efficacy of treatment, he showed that 40 per cent. of admissions were cured, and that the balance of admissions contained an enormous proportion of hopeless cases. Eighteen per cent. of all admissions are general paralytics or epileptics, 21·8 are aged fifty-five or over, 22 per cent. are admitted after a year's duration of the disease, and a further 13 per cent. after three months' duration. He advocated the establishment of two classes of hospitals; the first to form part of the machinery of county and borough asylums, into which all curable cases should be taken and kept for a certain time, unless, of course, recovery took place; the second to be erected near large towns supporting medical schools. Both are to be under the responsible charge of alienists, but both are to have the advantage of the services of general physicians. In the latter class of hospitals ample provision is to be made for systematic and clinical teaching, much on the lines of a general hospital. He gave considerable details as to the arrangements, the selection of cases, and the duties and relations of the staff.

A Bill for the establishment of a Dublin Hospital Board was issued on Friday night; it is practically an embodiment of the recommendations of the late Commission. It provides that all the hospitals shall be placed under the control of a Hospital Board, the constitution of which is intended to be representative of different religious denominations and of public bodies and institutions connected with hospital work or engaged in medical education. On the formation of the board the Government will place at their disposal out of the public funds a sum of £11,500, to be distributed in grants to such hospitals as comply with definite conditions. They must have not less than eighty beds occupied, and in five years the number must increase to 100, to be occupied not less than nine months of the year. The Rotunda and the Coombe Hospitals are exempted from these conditions. They are to be open to persons of all creeds without any distinction, and clergymen and members of all denominations are to be at liberty to visit at all hours of the day and night. They must employ nurses who have been trained or belong to a religious sisterhood. A scheme or schemes are to be prepared, after communication with the governing bodies, for the gradual abolition of the purchase system. There are various other details given regarding pensions, etc. There are several points in the Bill which would have called forth discussion had it not been rushed through Parliament just at the end of the session.

